# Statin-Induced Immune-Mediated Necrotizing Myopathy: A Case Report of a Rare and Underrecognized Cause of Progressive Weakness

**DOI:** 10.7759/cureus.96449

**Published:** 2025-11-09

**Authors:** Shivendra Tangutoori, Sai Subramanyam Kommineni, Dedeepya Gullapalli, Mythili kanthi Gudipati, Subramanya Shyam Ganti

**Affiliations:** 1 Internal Medicine, Appalachian Regional Healthcare, Harlan, USA; 2 Pulmonary and Critical Care Medicine, UMass Chan Medical School - Baystate, Springfield, USA; 3 Internal Medicine/Pulmonary Critical Care, Appalachian Regional Healthcare, Harlan, USA

**Keywords:** endocrine myopathy, immune-mediated necrotizing myopathy, lower limb weakness, statin-induced myopathy, statin induced necrotizing autoimmune myopathy

## Abstract

Statin-induced necrotizing autoimmune myopathy is a rare immune-mediated process that leads to muscle necrosis and occurs following exposure to statin therapy. The diagnostic clue for this disorder stems from the development of a proximal muscle weakness associated with the elevation of creatine kinase levels in association with the use of statin medication. Management mainly includes discontinuation of the statin medication, pursuing a tissue diagnosis, and early initiation of immunosuppressive therapy to preserve and regain muscle strength. We present the case of a 54-year-old patient with statin-induced necrotizing autoimmune myopathy who developed proximal muscle weakness a few months after initiation of statin therapy, was positive for anti-HMGCR (anti-hydroxy-methyl-glutaryl coA reductase) autoantibodies, and had evidence of muscle necrosis with minimal cellular infiltration on muscle biopsy. A definitive diagnosis can often be made without biopsy in the presence of positive anti-HMGCR antibodies and a compatible clinical presentation, though biopsy remains valuable for seronegative or atypical cases. He was treated with immunosuppressive therapy using intravenous immunoglobulins and was noted to have a good response. This case report highlights the importance of early recognition of statin-related adverse effects that require urgent evaluation and timely therapy to preserve and improve the functional status of a patient.

## Introduction

Statins are commonly used as lipid-lowering medications for the primary and secondary prevention of atherosclerosis-mediated vascular disorders. Considering their preventive strategy, long-term use of these medications is often required. Statin-associated muscle symptoms (SAMS), including myalgia with or without creatine kinase (CK) elevation, are common, affecting approximately 10-25% of patients in clinical practice, though most are not directly attributable to statins [1].

True statin-induced myopathy, characterized by significant CK elevation and rhabdomyolysis, is rare, with incidences of approximately 0.01-0.1% and <0.01%, respectively [2]. Asymptomatic hyperCKemia, where CK is elevated, may also occur. These conditions typically resolve within weeks to months after statin discontinuation. These forms of statin-induced myopathy vary distinctly from another clinical entity termed statin-associated immune-mediated necrotizing myopathy (IMNM), characterized by variable degrees of proximal muscle weakness, markedly elevated CK levels, evidence of muscle necrosis on histopathology, and anti-HMGCR (anti-hydroxy-methyl-glutaryl coA reductase) antibodies, suggesting an autoimmune-related phenomenon [3].

The incidence of IMNM is around one to three per 100,000 patient-years [4]. Unlike other statin-related muscle symptoms, anti-HMGCR IMNM often persists despite statin withdrawal and requires treatment with immunosuppressive medications to support recovery of muscle function.

## Case presentation

A 54-year-old man with a past medical history of hypertension, type 2 diabetes mellitus, prior stroke, hyperlipidemia, and long-standing atrial fibrillation on anticoagulation and amiodarone, presented with muscle weakness that started four to five weeks before the presentation. He had been having difficulty getting up from a seated position and lifting weights using his arms. He had no pain or skin rash accompanying the weakness. There was no evidence of bulbar or respiratory involvement, including dysphagia, dysarthria, or dyspnea, on clinical assessment. Statin therapy with atorvastatin (40 mg daily, high-intensity) was initiated five months prior as a preventive measure, considering his high risk for atherosclerotic cardiovascular disease.

On examination, there was no muscle tenderness. Proximal muscle strength was assessed using the Medical Research Council (MRC) scale: deltoids 3/5 bilaterally, hip flexors 3/5 bilaterally, and quadriceps 3/5 bilaterally. A chair-stand test showed an inability to rise from a seated position without using arms. Labs showed a marked elevation of CK of >7000 IU/L (reference, 22-198 IU/L). Renal function and TSH level were normal. Atorvastatin and amiodarone were discontinued due to the potential for amiodarone, a CYP3A4 inhibitor, to increase atorvastatin plasma levels, thereby exacerbating myotoxicity risk.

**Figure 1:**
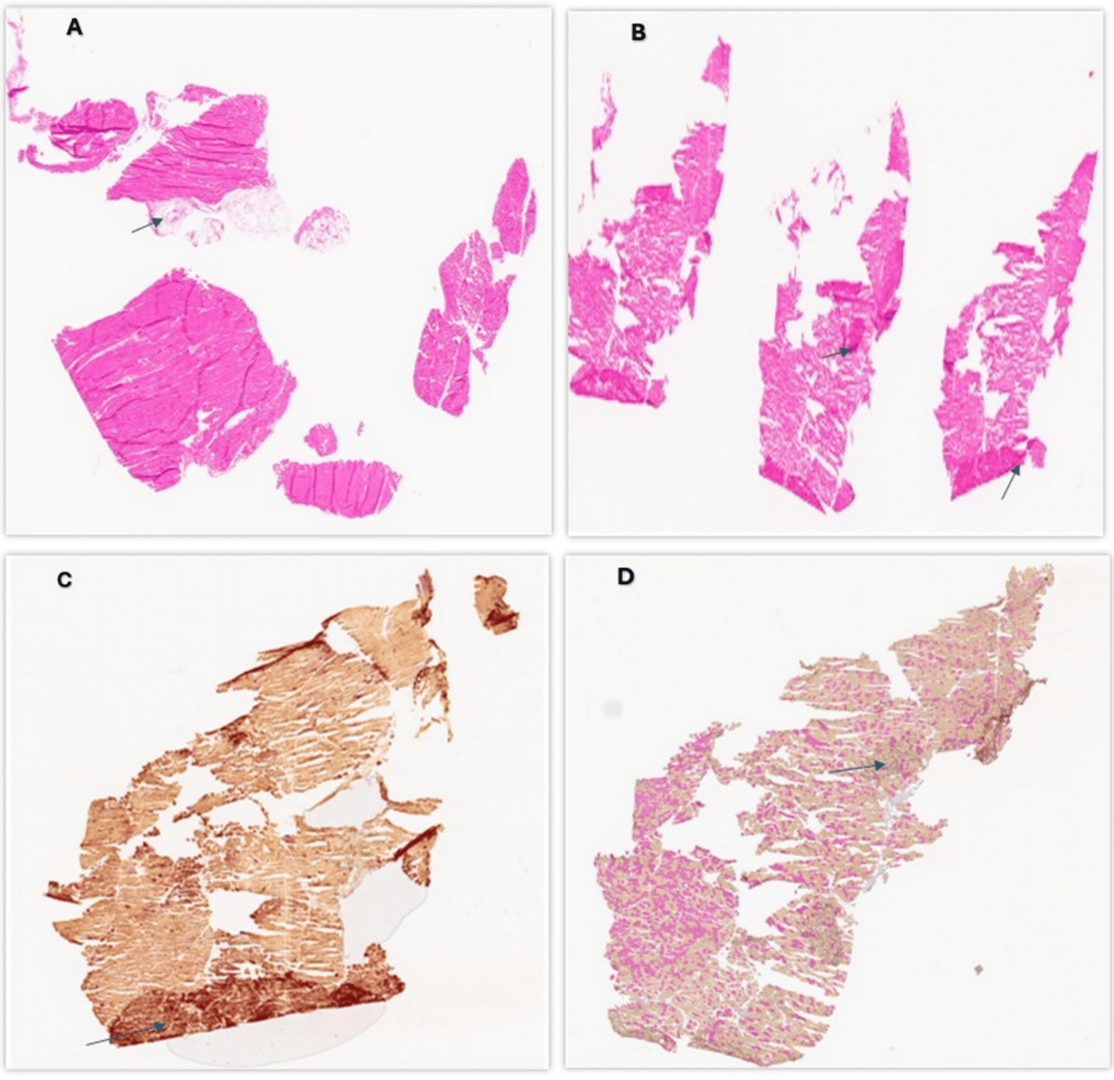
(A, B) H&E-stained section of the muscle biopsy, showing a necrotic fiber with some inflammatory cells; (C) Esterase staining highlighting the presence of macrophages associated with muscle fiber necrosis; (D) Immunohistochemistry reaction for fast myosin showed pattern of type I, IIA and IIB fibers. H&E: hematoxylin and eosin Image Credit: Subramanya Shyam Ganti

Autoimmune workup was negative for antinuclear antibody (ANA), rheumatoid factor, and anti-cyclic citrullinated peptide (CCP) antibodies. The muscle weakness continued to progress, limiting his functional status, and he eventually became wheelchair-bound. On the follow-up visit, further testing was pursued. Serum aldolase levels were elevated at >56 U/L. Anti-HMGCR was elevated at >200 U/mL (Table 1). There was high suspicion of statin-induced necrotizing myopathy. He was immediately referred to physical therapy rehabilitation to preserve his muscle function. A muscle biopsy was done of the patient’s quadriceps muscle, which demonstrated necrotic muscle fibers with scattered muscle atrophy and degenerative changes (Figure 1).

**Table 1:** Laboratory test results anti-HMGCR: anti-hydroxy-methyl-glutaryl coA reductase

Test	Patient Value	Reference Range
Creatine Kinase	>7000 IU/L	22-198 IU/L
Serum Aldolase	>56 U/L	1-7.5 U/L
Anti-HMGCR antibodies	>200 U/ml	less than 20 U/mL

A diagnosis of statin-induced IMNM was established. Following a referral to neurology for specialized care, he was deemed to be an ideal candidate for intravenous immunoglobulin (IVIG) therapy (1 g/kg every four weeks), due to severe muscle weakness and diabetes, which contraindicated high-dose steroids to avoid glycemic complications. Physical therapy was continued through the course of medical therapy. Serum CK levels were serially monitored and slowly downtrended. Table 2 summarizes the trends in CK levels and a modified Statin-Associated Muscle Symptom Clinical Index (SAMS-CI) score, adapted to include MRC-based strength assessment, over the treatment course.

**Table 2:** Trends in creatine kinase levels and functional status CK: creatine kinase; MRC: Medical Research Council; SAMS-CI: Statin Associated Muscle Symptom Clinical Index

Time Point	CK (IU/L)	Modified SAMS-CI Score (MRC-based, 0–15)*
Baseline	>7000	9 (deltoids 3/5, hip flexors 3/5, quadriceps 3/5)
4 weeks post-IVIG	4500	10 (deltoids 3+/5, hip flexors 3+/5, quadriceps 3+/5)
8 weeks post-IVIG	2000	12 (deltoids 4/5, hip flexors 4/5, quadriceps 4/5)

There was a clinical improvement noted gradually over a few weeks. He was able to ambulate using a walker and currently can walk a few steps without any form of support. He continues to follow up in the specialty care clinic and is continued on IVIG therapy and will be monitored closely for symptoms. At a follow-up eight weeks after IVIG initiation, MRC scores improved to deltoids 4/5, hip flexors 4/5, and quadriceps 4/5, with the patient able to perform one chair-stand using minimal arm support. He continues to follow up in the specialty care clinic, with a planned treatment of six months. Maintenance therapy will be continued if CK levels remain >500 IU/L or MRC scores are <4/5 in proximal muscle groups; tapering will be considered if CK normalizes (<198 IU/L) and MRC scores reach 5/5 with sustained functional independence.

The CARE (CAse REport)-compliant timeline of key clinical milestones is given in Table 3.

**Table 3:** CARE-compliant timeline of key clinical milestones MRC: Medical Research Council; IVIG: intravenous immunoglobulin; anti-HMGCR: anti-hydroxy-methyl-glutaryl coA reductase; INNM: immune mediated necrotising myopathy

Time Point	Milestone
Month 0	Initiation of atorvastatin (40 mg daily)
Month 4	Onset of proximal muscle weakness
Month 5	Initial CK measurement (>7000 IU/L)
Month 6	Anti-HMGCR antibody result (>200 U/mL)
Month 7	Right quadriceps muscle biopsy confirming IMNM
Month 8	Initiation of IVIG therapy (1 g/kg every 4 weeks)
Month 10	Functional recovery (ambulation with walker, MRC 4/5 in proximal muscles)

## Discussion

Statin-associated IMNM, corresponding to the anti-HMGCR subtype, is a rare adverse effect of statin therapy characterized by the acute or insidious onset of symmetric, proximal more than distal weakness. Dysphagia, dysarthria, or myalgia may also occur. Patients may have an underlying connective-tissue disorder (usually scleroderma or mixed connective-tissue disorder) or cancer (paraneoplastic necrotizing myopathy), or it may be idiopathic.

Statin-associated IMNM, primarily linked to anti-HMGCR antibodies, is a distinct subtype characterized by muscle fiber necrosis with minimal lymphocytic infiltration [5]. Unlike anti-SRP-associated or seronegative IMNM, which may relate to connective tissue disease or malignancy, statin-associated IMNM is typically identified by positive anti-HMGCR antibodies and a compatible clinical picture, often obviating the need for biopsy. Biopsy remains valuable in seronegative or atypical cases. Overall, two-thirds of IMNM patients have anti-SRP or anti-HMGCR antibodies, while one-third are seronegative; focal or generalized MHC class I upregulation is common [4].

The exact pathogenesis of NAM is not clear, but studies suggest that statins upregulate HMG-CoA (3-hydroxy-3-methylglutaryl-coenzyme A) autoantigen in regenerating muscle cells that, in turn, sustain an autoimmune response even after statin withdrawal, providing a mechanism for statin-induced IMNM [6]. Patients exposed to statins can develop IMNM at any time during the duration of statin therapy. Unlike self-limited statin-associated myalgia, which typically resolves within weeks to months after statin discontinuation, statin-induced IMNM is characterized by persistent muscle weakness and CK elevation despite statin withdrawal.

Immune-mediated necrotizing myopathies are principally humoral mediated. Patients with antibodies to HMGCR are divided into those who have a history of statin exposure and those who are statin-naïve (non-statin-induced necrotizing myositis). Non-statin-induced autoimmune necrotizing myopathy occurs in a subset of patients who are statin-naïve and have HMGCR antibodies. These patients tend to be younger, have high CK levels, and respond poorly to immunosuppressive therapy compared with statin-induced necrotizing myopathy [7]. IMNM, when associated with anti-signal recognition peptide antibodies, may be paraneoplastic, and laboratory and radiologic evaluation for malignancy should be considered. The SRP myopathies are typically subacute, aggressive, and have a relatively refractory course.

Patients with HMGCR antibodies who develop IMNM often require immunosuppressive therapy. There are no randomized controlled trials that support a treatment strategy, and most treatment options have been based on expert consensus and clinical case series. Although steroids are a cornerstone of immunosuppressive therapy for statin-induced IMNM, most patients require combination therapy with steroids and a second agent (e.g., methotrexate, azathioprine, or mycophenolate mofetil) or IVIG. In select cases, such as those with contraindications to steroids (e.g., diabetes) or severe weakness, IVIG may be used as first-line or monotherapy. In the Mayo Clinic cohort, patients who received two or more immunotherapeutic agents (glucocorticoids, steroid-sparing immunosuppressants like methotrexate, azathioprine, or mycophenolate mofetil, and IVIG) in the first three months of treatment were more likely to experience significant improvement [8]. Intravenous immunoglobulin therapy, 2 gm/kg per month, is commonly used in refractory cases that do not respond within 8-12 weeks of immunosuppressant medications. It is also used as an initial agent in patients with coexistent interstitial lung disease or myocarditis and for patients with severe relapses who have bulbar or respiratory muscle weakness [9]. Some patients may relapse after tapering immunosuppressive therapy and may require long-term immunosuppressive therapy. 

Managing dyslipidemia in patients with statin-induced IMNM requires careful consideration. Non-statin lipid-lowering therapies, such as ezetimibe or PCSK9 inhibitors, may be considered, with close monitoring for myopathy recurrence. Lipid management should be individualized based on cardiovascular risk and patient tolerance.

## Conclusions

Statin-induced immune necrotizing myopathy is a rare but potentially severe and debilitating complication of statin therapy. Unlike common SAMS, which are typically benign and resolve after statin discontinuation, anti-HMGCR IMNM causes persistent proximal weakness and elevated CK levels, requiring immunosuppressive therapy. Physicians should monitor patients for the development of muscle pain and/or weakness in patients on statin therapy, and prompt evaluation, including measurement of CK levels and anti-HMGCR antibodies, should be undertaken if symptoms suggestive of a myopathy arise. Early recognition and intervention are essential for a favorable outcome in these cases.
